# Awareness of Age-Related Gains and Losses in a National Sample of Adults Aged 80 Years and Older: Cross-Sectional Associations With Health Correlates

**DOI:** 10.1093/geroni/igad044

**Published:** 2023-05-15

**Authors:** Roman Kaspar, Hans-Werner Wahl, Manfred Diehl

**Affiliations:** Cologne Center for Ethics, Rights, Economics, and Social Sciences of Health, University of Cologne, Cologne, Germany; Network Aging Research, Heidelberg University, Heidelberg, Germany; Department of Human Development and Family Studies, Colorado State University, Fort Collins, Colorado, USA

**Keywords:** Developmental outcome, Institutionalized population, Representative survey, Subjective aging, Very old age

## Abstract

**Background and Objectives:**

Advanced old age is a life stage with a high likelihood of age-related loss experiences. However, little is known about remaining gain experiences and their relation with perceived losses and health correlates in community-dwelling very old adults. Moreover, virtually nothing is known in this regard about the experiences of individuals in long-term care settings. First, we strived to establish the normative course of age-related gains and losses in advanced old age. Second, we examined whether such gain/loss perceptions in advanced aging moderated health correlates.

**Research Design and Methods:**

Data came from the nationally representative survey “Old Age in Germany D80+” conducted in 2020/2021. The sample comprised 10 578 individuals aged 80–106 years, including 587 individuals in long-term care. We used the multidimensional Awareness of Age-Related Change (AARC) questionnaire and moderated regression to analyze associations with late-life health and functioning correlates.

**Results:**

Levels of AARC-Gains were higher than those of AARC-Losses across most of the age range. Long-term care residents showed more AARC-Losses and fewer AARC-Gains compared with community-dwelling adults and contributed significantly to an overall negative balance of more losses than gains in those aged 90 years or older. Regarding functional health and autonomy, negative age effects were amplified by AARC-Losses, but buffered by AARC-Gains. A more positive ratio of gains-to-losses predicted better health and functioning.

**Discussion and Implications:**

Findings suggest that the loss aspect of development in very late life might have been overstated in the existing literature. Perceived gains and losses are of critical importance for the understanding of health correlates in very old age.

**Translational Significance:** Little is known about perceived age-related gains and losses in the age 80+ population in general and in institutionalized older adults in particular. Reported experiences of gains even in advanced old age pointed to a possible psychological resource that still seems overlooked in practice but could, according to the findings of this study, help mitigate perceived age-related losses and their association with late-life health correlates.

## Background and Objectives

Several studies have shown that adults’ self-perceptions of aging (SPA) become increasingly negative as they age, with acceleration of the negativity trend during the fourth age (e.g., Diehl, Wettstein, et al., 2021; Kaspar et al., 2022; Kleinspehn-Ammerlahn et al., 2008; Miche et al., 2014). In fact, the ongoing discourse on the fourth versus the third age (e.g., P. B. Baltes & Smith, 2003; Wahl & Ehni, 2020) suggests that experiencing age-related gains in the fourth age may be quite limited due to the pronounced accumulation of biological, functional, cognitive, and social losses during this life stage. Still, life-span psychology also postulates that gains *and* losses co-occur across the entire life span, hence advanced old age should not be an exception (P. B. Baltes et al., 2006). In this article, we argue that compared to losses the empirical investigation of gains has received far less attention in very old age (Wurm & Schäfer, 2022). In fact, existing conceptual views of the “fourth age” (e.g., P. B. Baltes & Smith, 2003; Laslett, 1989) might have discouraged to some extent empirical research toward a more balanced view of gains and losses in very old age. As Gilleard and Higgs (2010) have argued, the fourth age is seen in the gerontological research community as “the unsuccessful but necessary counterpart to successful aging” (p. 121) and as “the collapse of the ‘third-age project’ where power, status, and citizenship can no longer be enacted (…)” (p. 121).

Specifically, the existing psychological, social, and epidemiological literature has over and over identified the objective losses coming with the fourth age; however, very little is known about perceived gains or strengths in community-dwelling very old adults and virtually nothing is known in this regard of very old individuals in long-term care settings. Given this scarcity of research on losses *and* gains, empirical research examining whether interindividual differences in self-perceptions of gains and losses in advanced old age may moderate health correlates is, to the best of our knowledge, nonexistent. A major problem in this area of inquiry has been that representative data on the fourth age have been limited, if not unavailable. Recent investments in Germany have changed this situation and have resulted in a national survey titled “Old Age in Germany D80+.”

### Previous Research on Perceived Gains and Losses in Old and Very Old Age

Some evidence suggests that SPA become increasingly negative as individuals age and particularly in advanced old age (Diehl, Wettstein, et al., 2021; Kaspar et al., 2022; Kleinspehn-Ammerlahn et al., 2008; Miche et al., 2014). However, only few longitudinal studies so far considered change in perceptions of both age-related gains (e.g., ongoing development) and losses (e.g., in the social or physical domain) as distinct self-perceptions across old age (Diehl, Wettstein, et al., 2021; Kaspar et al., 2022). Multi-dimensional scales that can provide independent evaluations of positive and negative experiences of the aging process may improve this situation but have not been used very much (Diehl & Wahl, 2010; Diehl et al., 2014). Diehl, Wettstein et al. (2021) examined the AgeCog scales (Wurm et al., 2007) in a longitudinal study and found in individuals 40 years and older from about age 65 an age-related increase in perceptions of physical and social losses. Comparatively, individuals started at about age 55 to report increasingly fewer perceptions related to ongoing development. Shenkin et al. (2014) applied the Attitudes to Aging Questionnaire (AAQ) in a sample of adults age 75 years and older and found generally positive attitudes for all AAQ-dimensions, that is, low psychosocial loss, high physical change, and high psychological growth. Brothers et al. (2017), using the 50-item Awareness of Age-Related Change (AARC) scale in a sample of adults age 40 and older, found correlations of chronological age with AARC-Gains of .14 and with AARC-Losses of .27, hence both associations were positive, although the strength of the association was significantly greater for AARC-Losses. Yet, research on SPA across the second half of life has remained inconsistent and limited.

In addition, the bulk of previous studies contained limited numbers of individuals in very old age. In particular, probability samples comprising large enough numbers of adults in very old age are scarce across the globe (Hansen et al., 2021; Kelfve et al., 2013; Kotter-Grühn et al., 2009; Stückler & Ruppe, 2015). Moreover, even studies dedicated to old and very old age sometimes fall short of fully representing hard to assess subgroups, such as those living in institutional settings, generally resulting in a positively biased picture of very old age (Kelfve et al., 2013; Schanze & Zins, 2019).

Recently, Kaspar et al. (2022) reported findings based on a sample of 912 individuals aged 80 years or older representative of the state of North Rhine-Westphalia (NRW), the most populous Federal state of Germany. The authors applied a 10-item short version of the AARC scale (AARC-10SF; Kaspar et al., 2019), differentiating between AARC-Gains and AARC-Losses, and reported decreasing levels of AARC-Gains and increases in AARC-Losses across a 2-year interval in very late life. Of note, associations of chronological age with change in AARC-Gains and AARC-Losses were found to be nonsignificant (*r* = −0.03, *p* = .429 and *r* = −0.02, *p* = .593, respectively), suggesting few age-related differences in self-perceived gains and losses in these very old individuals. Due to the sample of Kaspar et al. (2022) being representative only of the state of NRW, however, the degree of generizability of findings to other Federal states of Germany or even beyond Germany remained unclear.

### Self-Perceptions of Aging and Health Correlates in Very Old Age

A vast body of research has consistently documented that more negative SPA is associated with a range of unfavorable developmental outcomes, such as poorer physical and mental health, decreased cognitive functioning, including brain pathology, and increased all-cause mortality (for review, see Diehl et al., 2015; Diehl, Brothers, & Wahl, 2021; Westerhof et al., 2014; Wurm et al., 2017). The multidimensional AARC questionnaire (Brothers et al., 2019) has shown meaningful associations with health- and well-being related outcomes, such that higher gain- and lower loss-related perceptions co-varied quite consistently with better health and well-being (for an overview, see Sabatini, Silarova, et al., 2020). Furthermore, the AARC-Losses dimension has consistently shown stronger associations with developmental outcomes compared to the AARC-Gains dimension (for review, see Diehl, Brothers, & Wahl, 2021). For example, Dutt et al. (2018) showed that age-related losses, but not gains, were directly associated with changes in depression in midlife and old age. However, a more complex pattern of associations was found between depression and lack of AARC-Gains, with the latter amplifying depression when combined with low levels of accommodative strategies.

Also of note in this context, Wurm and Schäfer (2022) recently found, based on data from the multidimensional AgeCog Scale (Wurm et al., 2007), that self-perceived gains, but not self-perceived losses predicted longevity across a 23-year observational period. Similarly, significant unique effects of AARC-Gains on late-life survival have been shown in a very old sample across a 2-year period over and above competing socio-behavioral determinants of longevity, such as perceived control or positive appraisal of life (Kaspar et al., 2021).

Hence, reliably identifying individuals in their fourth age who report gain-related perceptions of their aging process even under adverse circumstances or identifying individuals with particularly dysfunctional negative perceptions of their own aging, may help to develop and offer tailored interventions for successful aging.

In summary, three conclusions may be derived from the existing research: First, studies on very old individuals and individuals in long-term care settings, permitting a robust testing of the associations between SPA gains and losses and health-related outcomes have remained very rare (for exceptions, see Kaspar et al., 2021, 2022). Second, the potentially differential effects of perceived gains and losses on health correlates need more attention in adults in advanced old age. Furthermore, no study has so far directly assessed the effect of the ratio of gains-to-losses regarding SPA in terms of predicting late-life correlates. Third, very old individuals in long-term care settings tend to be under-represented in many large-scale aging studies. Therefore, it is important to extend the empirical testing of SPA-related gain and loss dynamics also to institutionalized older adults. Findings from such research may have the potential to influence public health initiatives that focus on the implementation of interventions with individuals in their fourth age in home health care as well as in nursing home settings (Diehl, Rebok, et al., 2021; Klusmann et al., 2012).

### Research Aims and Hypotheses

First, given that only limited data are available, this study examined the extent to which gain- and loss-related aging awareness exists in those 80 years and older, including in individuals in long-term care. By doing so, we were able to take advantage of the largest population-based sample ever generated with adults in very old age. We expected to confirm the two-dimensional structure of the established AARC-10 Short Form (Kaspar et al., 2019, 2021, 2022) and measurement equivalence across modes of administration (i.e., self-administered vs. administered in phone interviews). Based on the conceptual literature on the specific challenges of the fourth age (M. M. Baltes, 1996, 1998; Heckhausen et al., 2013) as well as previous findings for age-related trajectories of AARC, we expected a greater increase in AARC-Losses compared to the expected decrease in perceived AARC-Gains across the very late life period, resulting in a less favorable ratio of gains-to-losses in the oldest old. Finally, we expected for very old individuals in long-term care higher levels of perceived AARC-Losses and lower levels of perceived AARC-Gains as compared to community-dwelling individuals.

Second, we examined the cross-sectional associations of the AARC-Gains and AARC-Losses scales with key health correlates in advanced old age. We expected that higher AARC-Gains would be associated with more favorable developmental correlates, such as greater independence in activities of daily living (ADLs), and greater perceived autonomy. Moreover, we expected that the balance of perceived age-related gains to losses, as a potential indicator of successful coping with late-life challenges, would also be associated with health and functioning correlates. Specifically, we assumed that self-perceived losses would be associated with poorer levels of health and functioning and that this association would be strongest in the absence of self-perceived gains.

Finally, we expected AARC to account for differences in the strength of association between chronological age and health and functioning outcomes. In general, we expected worse health conditions to be associated with a higher chronological age even in this sample of already very old adults. Specifically, we expected a stronger age-associated decrease in health/functioning in individuals who reported more AARC-Losses; in parallel, we expected weaker age health/functioning associations in individuals who reported more AARC-Gains.

## Research Design and Methods

### Transparency and Openness

We report how the sample size was determined, describe all data exclusions, manipulations, and all measures in the study, and follow the Journal Article Reporting Standards (JARS; Appelbaum et al., 2018). All data and research materials are available from the German Centre of Gerontology research data repository (Albrecht et al., 2023). The study was approved by the ethical board of the medical faculty at the University of Cologne (Protocol #: 19-1387_1). This study’s design and analyses were not preregistered. Data were analyzed using SAS 9.4 (SAS Institute, 2013) and Mplus 8.7 Software (Muthén & Muthén, 1998–2017). Analysis code and output are available from the Open Science Framework repository at https://doi.org/10.17605/OSF.IO/9SFT8.

### Participants and Procedures

Data came from the national survey titled “Old Age in Germany (D80+).” The sample is representative of the population 80 years and older and was derived in a three-step sampling process (i.e., states, community, individual) with proportional-to-size sampling at the level of all 16 Federal states of Germany and the community level. Persons in older age groups (i.e., 84 years and older) and men were oversampled to allow for meaningful subgroup analyses. Sample size was determined based on recruitment rates realized in a preceding state-wide representative survey in this age segment (Hansen et al., 2021) and with the aim of achieving comparable precision in estimating population values. The survey employed a sequential mixed-mode design (i.e., paper-and-pencil survey followed by a phone interview) because of limited face-to-face access during the pandemic (November 2020 to December 2021). The final sample comprises 10 578 individuals, 10 360 of which returned a paper-pencil questionnaire in the initial stage and 218 additional respondents who were included only via computer-assisted telephone interviews (CATI) in the subsequent stage of recruitment. A total of 587 respondents resided in nursing homes. Mean age of the realized sample at the time of the interview was 86.9 years (standard deviation [*SD*] = 4.4 years; range: 80.9–106.0 years).

### Sample Characteristics

Due to the disproportionate sampling design, the realized sample included similar proportions of men and women, as well as a high share of individuals in each of the three design age groups 80–84, 85–90, and 90 years or older (Supplementary Table 1). The level of education was less advantaged than in available nonrepresentative samples of very old adults (Generali Zukunftsfonds, 2014). Individuals living in nursing homes accounted for less than 6% of the unweighted sample. The overall sample had an average of 4.3 currently treated health conditions and a low level of depressive symptoms. With respect to developmental correlates, respondents reported a high degree of perceived autonomy with an average of 3.4 scale points on the four-category item and independence in IADL with an average of 1.4 scale points on the 0–2 points scale. The sequential mixed-mode design aimed at including more vulnerable individuals (i.e., individuals who did not return a written questionnaire) via phone interviews. CATI respondents included a higher share of women, more individuals from nursing homes, and those with lower levels of education. Differences with respect to developmental correlates in the health and well-being domain suggested significantly more depressive symptoms and higher multimorbidity in those participants recruited via personal phone contact. In CATI interviews, where the Demtect (Kalbe et al., 2004) has been used as a screening tool for mild cognitive impairment (MCI), the estimated prevalence of MCI and early stages of dementia was comparable with expectations from epidemiological studies in Germany (Brijoux & Zank, 2022). In sum, we assume that the inclusive study design resulted in a less positively biased sample and, therefore, in a more realistic representation of individuals in very old age.

### Measures

Participants’ socio-demographic information, level of education (Unesco, 2015), and information regarding living arrangements were assessed via a standardized personal background questionnaire.

#### Awareness of age-related change

The 10-item short form of the AARC scale (AARC-10SF; Kaspar et al., 2019) was used as a brief measure of individuals’ SPA. The AARC-10SF is multidimensional in capturing adults’ self-perceptions of age-related gains and losses across five behavioral domains: Health and physical functioning, cognitive functioning, interpersonal relations, social-cognitive and social-emotional functioning, and lifestyle and engagement (see Supplementary Table 2 for item wording). Evidence for concurrent, discriminant, and predictive validity of the AARC-Gains and AARC-Losses scales has been reported in different countries (Kaspar et al., 2019, 2021, 2022; Neri et al., 2021; Sabatini, Ukoumunne, et al., 2020). Like for the long form of the questionnaire (Brothers et al., 2019), the two-factor solution was confirmed for the AARC-10SF, using confirmatory factor analysis and independent samples (Kaspar et al., 2019; Sabatini, Ukoumunne, et al., 2020).

#### Late-life health and functioning correlates

The number of self-reported currently treated health conditions was used as an indicator of *multimorbidity*. Hence, this measure refers to a subset of medical conditions with high salience for the individual in everyday life. This index was modified from the Self-Administered Comorbidity Questionnaire (SCQ; Sangha et al., 2003) to include medical conditions particularly relevant in old age (Wiest et al., 2014). The 19 conditions are heart disease (e.g., insufficiency), heart attack, hypertension, respiratory or lung disease, diabetes, gastrointestinal disease, kidney disease, liver disease, and hemophilia (e.g., anemia), cancer, mental disease (e.g., phobia, depression), bone and joint disease (e.g., osteoporosis, arthrosis, arthritis), back pain, urinary disorder, insomnia, hearing impairment, visual impairment, neurological disease (e.g., Parkinson’s, dementia), and stroke.

Adults’ self-reported performance on Instrumental Activities of Daily Living (IADL; Lawton & Brody, 1969) was used as a measure of *independence in everyday functioning*. In this paper, we use seven items representing IADL (e.g., preparing meals, handling finances) with response options 0 = “not possible without help,” 1 = “some help needed,” 2 = “no help needed.” Reliability of the IADL scale in the current sample was high (α = 0.92). *Perceived autonomy* was assessed using the single item “I live my life according to my own preferences” with response options 1 = “not at all” to 4 = “very much.”

*Depressive symptoms* during the past 2 weeks were assessed with the short form of the Depression in Old Age Scale (DIA-S4; Heidenblut & Zank, 2020). The four items asked for the occurrence of lack of motivation, feelings of sadness, worrying, or inability to enjoy life. Favorable diagnostic properties of the DIA-S4 have been shown for distinguishing groups of depressed and nondepressed geriatric inpatients (Heidenblut & Zank, 2014, 2020). Scale consistency in this sample was acceptable (α = 0.67).

### Plan of Analysis

First, we describe levels and heterogeneity of the subjective experience of aging based on the AARC measure across age groups in old age and in individuals in private versus institutional dwellings. We used confirmatory factor analysis (CFA) to estimate the conceptually expected dimensions of AARC-Gains and AARC-Losses. AARC factor scores were effect-scaled for purposes of identification, most flexible parameter constraints in measurement invariance (MI) testing, and straightforward interpretation and comparison of scale scores (Little et al., 2006). We employed multi-group CFA with equality constraints to test for MI between questionnaire and CATI modes of administration. Second, we tested both main effects and potential moderation effects of AARC-Gains or AARC-Losses on the association between chronological age and health- and functioning-related outcomes, using hierarchical linear regression with interaction terms. Predictors were mean centered to increase interpretability of moderation effects. We used Johnson–Neyman plots (Johnson & Fay, 1950) to identify the levels of AARC-Gains and AARC-Losses at which the moderation effects occurred.

All analyses used weights to correct for the disproportional sampling design (i.e., oversampling of men and older age groups), nonavailability of phone numbers for follow-up contacting, and survey nonresponse (Prussog-Wagner et al., 2022). Computed standard errors of parameter estimates were corrected for the clustering due to the multistage sampling procedure.

## Results

### Psychometric Properties of the AARC-10SF

A detailed description of responses to the AARC-10SF items is given in Supplementary Table 2. Item nonresponse was higher for items related to gains in the RELSHP and COGN domains, possibly due to the rather complex evaluations needed to respond to these items. Overall, rates of nonresponse were acceptable given that most responses were collected via mailed questionnaires. Likewise, evidence of floor or ceiling effects was very limited, with a maximum of 20.2% answering “not at all” to the RELSHP- item “dependent on help” and 19.3% endorsing the “very much” category with item ENGAGE- “limit activities.” With the exceptions of the latter item and item PHYS- “less energy,” lower levels were generally observed in the losses domain compared to the gains domain. Responses with respect to age-related experiences of increasing dependency on others (i.e., RELSHP-) were more heterogeneous (*SD* = 1.32) than experiences of other age-related developments.

A two-factor configural multi-group CFA with freely estimated factor loadings and item intercepts for AARC-10SF responses from self-report questionnaires and CATIs showed an acceptable model fit (Supplementary Table 4). Consecutively more restrictive models with parameters constrained equal for questionnaire and CATI respondents did not significantly increase model misfit. Hence, analyses established scalar MI, allowing for direct comparison of scale scores derived from paper-and-pencil questionnaires and CATI assessments. Composite reliability (MacDonald’s ω, Revelle & Zinbarg, 2009) in the current sample was 0.71 and 0.86 for the gains and losses subscales, respectively, suggesting somewhat greater heterogeneity in the experience of age-related gains.

### Normative Patterns of Age Differences in Perceived Age-Related Gains and Losses

The estimated level of age-related gains was higher (3.21) than that of age-related losses (2.91) in the total sample (Table 1). With effect-coding of latent factors and nonoverlapping estimated confidence intervals for gains and losses mean scores (i.e., 3.20–3.23 vs. 2.89–2.93), this difference across subscales was statistically significant. Of note, in terms of assessment mode, significantly lower levels of AARC-Gains were reported in CATI interviews compared to responding to mailed questionnaires. Hence, excluding the somewhat more vulnerable group that failed to send back a questionnaire would have resulted in biased estimates of perceived age-related gains in very old adults. Gender differences were small but statistically significant with respect to both slightly lower levels of perceived gains (3.19 vs 3.25) and somewhat higher levels of age-related losses reported by women compared to men (2.99 vs 2.79). Moreover, in line with expectations from bivariate associations between study variables (Supplementary Table 3), low levels of education and living in an institution were associated with less AARC-Gains and more AARC-Losses.

**Table 1.** Perceived Age-Related Gains and Losses in Very Old Age by Subpopulation

Variable	*N*	AARC-Gains	AARC-Losses
		*M* (95% CI)	*M* (95% CI)
Survey mode			
Questionnaire	10,359	3.22 (3.20–3.23)	2.91 (2.89–2.93)
Phone interview	110	**2.92 (2.79–3.06)**	2.87 (2.70–3.04)
Region			
East Germany	2,306	3.24 (3.22–3.27)	2.91 (2.87–2.95)
West Germany	8,163	3.21 (3.19–3.22)	2.91 (2.89–2.93)
Gender			
Men	3,982	3.25 (3.24–3.27)	2.79 (2.76–2.81)
Women	6,486	**3.19 (3.17–3.21)**	**2.99 (2.96–3.01)**
Age group			
80–84 years	6,177	3.27 (3.26–3.29)	2.73 (2.71–2.76)
85–89 years	2,823	**3.18 (3.15–3.20)**	**3.05 (3.01–3.08)**
90 years or older	1,469	**3.05 (3.02–3.08)**	**3.39 (3.35–3.44)**
Education (ISCED)			
Low	2,379	3.12 (3.10–3.15)	3.09 (3.05–3.13)
Mid	6,008	**3.23 (3.21–3.24)**	**2.87 (2.85–2.90)**
High	1,748	**3.31 (3.28–3.33)**	**2.77 (2.73–2.81)**
Living arrangement			
Private dwelling	9,410	3.25 (3.24–3.26)	2.84 (2.82–2.86)
Institutional setting	1,058	**2.89 (2.83–2.95)**	**3.55 (3.47–3.63)**
Total	10,469	3.21 (3.20–3.23)	2.91 (2.89–2.93)

*Notes*: AARC = Awareness of Age-Related Change; ISCED = International Standard Classification of Education. Weighted data. Subgroup differences statistically significant at the *p* < .05 level (nonoverlapping 95% confidence intervals) are given in bold font.

**Figure 1.**
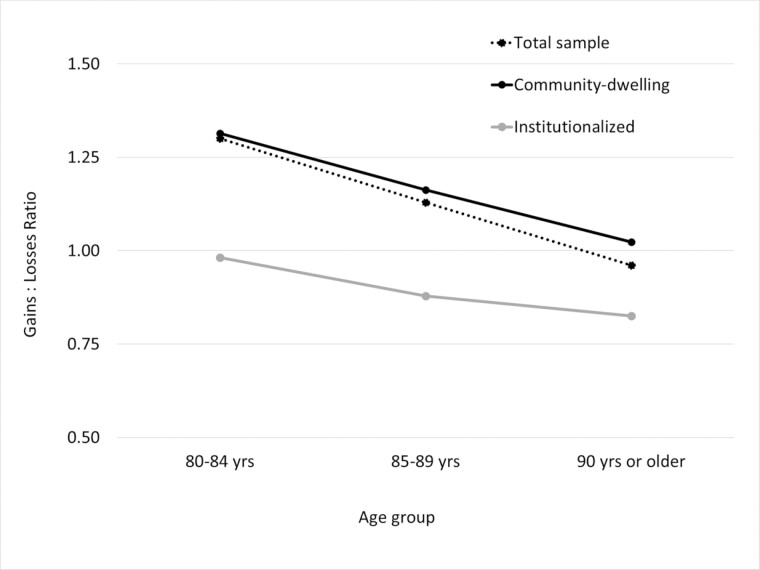
Awareness of age-related change (AARC) gains-to-losses ratio in community-dwelling and institutionalized older adults across age groups.

Differences between age groups were found for both AARC-Gains and AARC-Losses, with increasingly unfavorable perceptions of one’s own aging reported in older age groups (Table 1). However, the gains-to-losses balance was positive for younger age groups and only flipped toward a dominance of losses for those beyond the age of 90 (see also Figure 1). More fine-grained age-specific reference data which may serve as norm values for the AARC-10SF are given in Supplementary Figures S1 and S2 expressed as rank percent values from the 2.5th to the 97.5th percentile. These figures also indicate that heterogeneity of SPA was retained in very old and oldest age and corresponded with small negative and moderate positive bivariate associations of chronological age with AARC-Gains and AARC-Losses, respectively (Supplementary Table 3).

As expected, individuals in long-term care settings reported significantly lower levels of perceived gains and higher levels of losses compared to individuals in private homes (Table 1 and Figure 1). That is, very old adults in institutions showed a preponderance of perceived age-related losses over gains in general that became even less favorable across age groups. Important for evaluating trajectories in the full sample, oldest adults in nursing homes contributed substantially to the reversal of the balance of gains-to-losses observed for the total sample at the age of 90 or older. Exploratory sensitivity analyses suggested that significantly higher levels of losses and lower levels of gains in nursing home residents were not restricted to the PHYS or COGN domains but generalized across all five behavioral domains (Supplementary Table 5). However, levels of item RELSHP+ “appreciate people and relationships much more” were not significantly different in private settings and in institutions.

### Association between AARC and Health Correlates of Aging

Chronological age was a significant predictor of interindividual differences in functional health and perceived autonomy, with less favorable outcomes in oldest-old individuals. No association was found between chronological age and late-life depression and multimorbidity. Both AARC-Gains and AARC-Losses consistently contributed to the explanation of observed differences in all health- and function-related outcomes in main effect models that were conducted as the first step of hierarchical regression analysis (Table 2, Model 0). Specifically, higher self-perceived AARC-Losses were associated with greater multimorbidity, poorer functional health, lower autonomy, and more depressive symptoms. In contrast, higher self-perceived AARC-Gains were associated with better functional health, greater autonomy, and fewer depressive symptoms, but also with greater multimorbidity. The main effects model accounted for nearly half of the observed variance in IADL dependency, and 15% of interindividual differences in multimorbidity, representing high and moderate model determination (Cohen, 1988).

**Table 2.** Prediction of Developmental Outcomes by AARC-Gains, AARC-Losses, Chronological Age, and Their Interactions

Standardized regression coefficient	Main effects (Model 0)				Interaction effects (Model 1)^a^			
	Age	Gains	Losses	*R*²	Age × gains	Age × losses	Gains× losses	Δ*R*²
Developmental outcome (dependent)								
Multimorbidity (0–19)	<0.01 n.s.	0.06***	0.39***	15.0	0.04**	−0.06***	0.04**	0.9
Functional health (IADL 0–2)	−0.25***	0.15***	−0.54***	49.2	0.02 n.s.	−0.03***	0.04**	0.3
Autonomy (1–4)	−0.07***	0.20***	−0.38***	23.1	0.04*	−0.07***	0.04*	1.0
Depressive symptoms (DIA-S4, 0–4)	<−0.01 n.s.	−0.08***	0.49***	25.1	0.02 n.s.	<0.01 n.s.	−0.03**	0.1

*Notes*. AARC = Awareness of Age-Related Change; IADL = Instrumental Activities of Daily Living; DIA-S4 = Depression in Old Age Scale with four items. Weighted data.

^a^Predictors have been centered at the grand mean.

***p < .001. **p < .01. *p < .05. n.s. *p* ≥ .05.

Both AARC-Gains and AARC-Losses explained differences in outcomes in combination with age effects, although interactions were found only for physical health and function-related outcomes (Table 2, Model 1); for depressive symptoms, no moderation of the negligible effects of chronological age by perceived AARC-Gains or AARC-Losses was observed. Regarding IADL independence and autonomy, negative age effects (i.e., worse developmental outcomes with increasing chronological age) were amplified by AARC-Losses. Perceived AARC-Gains showed a significant buffering effect on age-associated decline only with respect to autonomy. Examination of confidence bands for estimated age effects across the continuum of moderator values provided additional insights (Supplementary Figure 3, left column). First, negative effects of higher chronological age on IADL independence were amplified along the continuum of increasing perceived AARC-Losses, but buffered by increasing perceived AARC-Gains. Self-perceived autonomy was no longer related to chronological age when individuals reported higher-than-average AARC-Gains. Furthermore, reporting low levels of AARC-Losses was associated with significant positive associations of chronological age with perceived autonomy, whereas associations with age became increasingly negative at higher-than-average levels of AARC-Losses. With respect to the age-multimorbidity link, lower-than-average AARC-Losses predicted an increasing positive association of multimorbidity with chronological age, whereas high levels of AARC-Losses were linked with fewer treated diseases with increasing age. A reversed pattern appeared for AARC-Gains, with lowered multimorbidity in older adults (i.e., a negative age-multimorbidity association), when levels of AARC-Gains were particularly low and higher multimorbidity in older adults with higher-than-average perceived AARC-Gains.

In addition to these associations between chronological age and AARC-Gains/Losses experiences, inter-individual differences in each late-life health correlate considered in this study were explained by specific gains-to-losses constellations (see Table 2, Model 1). That is, the level of concurrent age-related gains predicted if (i.e., regarding statistical significance) or to what degree perceived age-related losses resulted in outcomes, and vice versa. For functional health and autonomy, concurrent AARC-Gains buffered the adverse effect of AARC-Losses, whereas the latter increased the impact of concurrent AARC-Gains on these late-life functioning outcomes (Supplementary Figure 3, right column). The positive association between AARC and number of treated diseases increased in general with the co-occurrence of both gains *and* losses. At particularly low levels of AARC-Losses, however, associations between AARC-Gains and multimorbidity were nonsignificant. Finally, perceived AARC-Gains significantly buffered the adverse effects of concurrent AARC-Losses on depressive mood for all but the lowest levels of perceived age-related losses.

## Discussion and Implications

This study conducted a large-scale inquiry into the associations of self-perceptions of aging, assessed as AARC-Gains and AARC-Losses, with late-life health and functional outcomes. A major rationale behind the study was that advanced old age comes with the stigma of being only characterized by loss, although reliable data on how very old age is perceived by individuals are missing. This is the case for primarily two reasons. First, samples of individuals in advanced old age of desirable size and quality are rare, when it comes to SPA. Second, self-perceived gains in advanced old age require a more refined and balanced assessment vis-à-vis self-perceived losses. To improve this situation, the present study applied the brief and versatile AARC-SF scale with five items each to assess awareness of age-related gains and losses, respectively.

Towards this end, we confirmed in a large and heterogeneous sample of adults aged 80 and older (i.e., the fourth age) that the AARC-10SF is a reliable assessment of two distinct factors of their SPA: perceived age-related gains and perceived age-related losses. Similar to studies that used different single assessment modes (e.g., written questionnaire: Brothers et al., 2019; Kaspar et al., 2019; Sabatini, Ukoumunne, et al., 2020, [computer-assisted] personal interview: Kaspar et al., 2021, 2022; Neri et al., 2021), this study found the assessment to be statistically equivalent across written questionnaires and CATI, supporting the additional versatility of this brief measure.

Importantly, we found high levels of AARC-Gains throughout advanced old age. In fact, there were even higher levels of AARC-Gains compared with AARC-Losses. Although these findings corroborate previous findings in a state-level sample of very old adults (Kaspar et al., 2022), such high levels of perceived gains and moderate levels of perceived losses in the fourth age are intriguing given the high prevalence of multimorbidity and functional limitations in this sample. This may suggest an under-appreciated capability of very old adults to recognize opportunities for experiencing new growth in very old age or at least to retain previous age-related gains far into the fourth age. Explanations might lie in flexibility in goal adjustment maintained even in very old age (Brandtstädter, 1999) and/or the ongoing capacity for selective optimization with compensation (Freund et al., 2017).

The current study provided age-specific norm values (presented as rank percent) from a national sample and will help to reliably interpret the relative position of any specific individual with respect to their perception of age-related gains and losses assessed with the AARC-10SF. In contrast to ARRC-Gains, more pronounced differences were found across age groups for AARC-Losses and significantly more losses were found for older adults in nursing homes. Still, many adults in their fourth age showed only moderate levels of perceived age-related losses (i.e., interquartile range 2.3–3.5), suggesting that some objective losses might not be attributed to growing older (as the AARC measure requires). In-depth analysis of both the effects of life events on AARC and potential developmental shifts in how items of the AARC-10SF are interpreted and answered may foster a better understanding of these surprising effects. With respect to life events, Rupprecht et al. (2022) recently showed in a sample aged 16–96 years that the cumulative experience of health events was associated with greater awareness of both age-related losses and age-related gains.

In line with expectations from developmental theory and existing findings (Heckhausen et al., 1989; Smith, 2003; Wahl & Ehni, 2020), we found the ratio of age-related gains to losses to grow less favorable across age groups, but even the oldest individuals retained substantial levels of perceived age-related gains. It was only for those aged 90 years or older that more AARC-Losses were reported on average than AARC-Gains, and this finding could be traced back to a very unfavorable gains-losses-balance in the institutionalized oldest old.

Further, based on this study’s findings of significant, differentiated, and dynamic associations between AARC and health correlates and previous evidence on the malleability of SPA (Beyer et al., 2019; Brothers & Diehl, 2017; Diehl, Rebok, et al., 2021; Klusmann et al., 2012), the AARC-10SF may prove useful in the arena of large-scale health monitoring or counseling to identify and support individuals at high risk of adverse late-life health outcomes. The finding that AARC-Gains were positively related to treated health conditions may indicate greater health competency and lower risk of underdiagnosis of health issues in those reporting more positive SPA. In addition, evidence of moderating effects of AARC on the association between chronological age and late-life health correlates might offer multiple starting points for intervention. First, because the link between chronological age and IADL independence and perceived autonomy was stronger in older adults that attributed experienced losses to aging, interventions may target health mechanisms beyond (biological) aging to increase active coping efforts. For example, misconceptions that health conditions are causally driven by chronological age may frustrate older adults’ motivation to seek treatment or consider lifestyle interventions. In line with this interpretation, higher-than average AARC-Losses were associated with a negative age multimorbidity association; that is, fewer treated health conditions with increasing age. Second, results showed that SPA may render chronological age irrelevant for perceived autonomy as long as there are higher-than-average AARC-Gains.

We found a unique contribution of the gains-to-losses ratio to account for differences in health correlates in addition to chronological age and SPA. Most prominently, higher levels of AARC-Gains were able to mitigate the negative associations between AARC-Losses and functional health and perceived autonomy. At the same time, AARC-Gains were irrelevant for most late-life health outcomes at very low levels of concurrent AARC-Losses. Hence, interventions may be tailored to focus on the promotion of perceived gains or the reduction of perceived losses, depending on the highest prospect of outcome modification at the given ratio of gains-to-losses.

Substantive associations with background variables corroborated previous validity evidence for the AARC-10SF. More specifically, individuals in nursing homes and with lower levels of education reported lower AARC-Gains and higher AARC-Losses. To the best of our knowledge, no direct comparison of SPA in very old adults living in private housing and institutions has been reported before, and the finding of a particularly unfavorable balance of gains-to-losses in nursing home residents is in line with theory about depletion of coping capabilities (Heckhausen et al., 2013).

## Limitations

In light of limited feasibility of face-to-face interviews during the pandemic, the D80+ study employed a sequential mixed-mode design. Because phone interviews were more successful than paper-and-pencil questionnaires in recruiting more vulnerable very old individuals (e.g., from nursing homes), their combination helped to increase the representativity of the sample. However, it is unlikely that the subsample of individuals living in institutions is completely representative of the population of nursing home residents, because even combined strategies of recruitment may not have succeeded to include individuals with the most severe impairments (e.g., advanced cognitive decline or sensory loss).

Moreover, findings are based on a large-scale, representative national sample, but only refer to individuals in fourth age, probably limiting the scope to only a subset of life experiences relevant to SPA from a life-span perspective (Rupprecht et al., 2022).

Finally, we note as a limitation of the current study that with cross-sectional data, associations with developmental outcomes cannot be interpreted as directional or causal.

## Conclusion and Future Directions

Based on our findings of direct effects of SPA on late-life outcomes, robust additional effects of the gains-losses-ratio, and findings that SPA explained differences in the association between chronological age and late-life health correlates, we conclude that the relatively “new life stage” of very old age itself may be characterized by a rather complex dynamic of perceived gains and losses in retaining health and functioning. Baltes and Smith (2003) referred to this situation as an “incomplete biocultural architecture of ontogeny for the oldest ages” (p. 130). Associations with health and functioning outcomes were different for AARC-Gains and AARC-Losses as well as the ratio between them, speaking to the benefit of assessing adults’ SPA in a multi-dimensional way.

Currently, we are not aware of any SPA-related measure in any of the existing measurement batteries, such as the CERAD, or in standard geriatric assessment tools, despite the fact that adults’ SPA has emerged as a robust predictor of a whole host of developmental outcomes (e.g., Westerhof et al., 2014). In summary, the availability of the AARC-10SF as a brief and robust measure of SPA, and the availability of age-specific norm values can help identify individuals who may profit from late-life health monitoring, counseling, or intervention to foster awareness of potential growth and protect favorable developmental outcomes far into very old age.

## Supplementary Material

igad044_suppl_Supplementary_Material
