# Supplementary material for: Awareness of Age-Related Gains and Losses in a National Sample of Adults Aged 80 Years and Older: Cross-Sectional Associations With Health Correlates
Source: Innov Aging. 2023 May 15;7(4):igad044. doi: 10.1093/geroni/igad044 (PMC10317143; doi:10.1093/geroni/igad044)
Supplement: igad044_suppl_Supplementary_Material [file igad044_suppl_supplementary_material.docx]

Online Supplementary Material

Supplementary Table S1.

*Socio-Demographic Characteristics, Health Status and Developmental Outcomes in the Sequential Mixed-Mode Sample.*

| Variable | Total Sample  (N=10,578) | Questionnaire (n=10,360) | Phone interview (n=218) |
| --- | --- | --- | --- |
|  | % or M ± SD | % or M ± SD | % or M ± SD |
| Region |  |  |  |
| East Germany | 20.0% | 19.9% | 22.0% |
| West Germany | 80.0% | 80.1% | 78.0% |
| Gender |  |  |  |
| Men | 48.3% | 48.6% | 33.9% |
| Women | 51.7% | 51.4% | 66.1% |
| Education (ISCED) |  |  |  |
| Low | 22.2% | 22.2% | 25.2% |
| Mid | 59.8% | 59.8% | 59.4% |
| High | 18.0% | 18.0% | 15.4% |
| Age group |  |  |  |
| 80-84 yrs | 47.6% | 47.6% | 49.1% |
| 85-89 yrs | 31.6% | 31.6% | 28.4% |
| 90 yrs or older | 20.8% | 20.8% | 22.5% |
| Living arrangement |  |  |  |
| Private dwelling | 94.4% | 94.5% | 90.4% |
| Institutional setting | 5.6% | 5.5% | 9.6% |
| Multimorbidity (0-19) | 4.3 ± 2.5 (0-17) | 4.3 ± 2.5 (0-17) | 3.6 ± 2.5 (0-11) |
| Functional health (IADL 0-2) | 1.4 ± 0.6 | 1.4 ± 0.6 | 1.3 ± 0.7 |
| Autonomy (1-4) | 3.4 ± 0.8 | 3.4 ± 0.8 | 3.3 ± 0.9 |
| Depressive symptoms (DIA-S4, 0-4) | 1.3 ± 1.3 | 1.3 ± 1.3 | 1.1 ± 1.2 |

Note. ISCED = International Standard Classification of Education; IADL = Instrumental Activities of Daily Living; DIA-S4 = Depression in Old Age Scale with four items. Unweighted data.

Supplementary Table S2.

*Perceived Age-Related Gains and Losses at the Domain-Level.*

| Domain^a^ | With my increasing age, I realize that … | Total sample (n = 10,578) | | | | | |
| --- | --- | --- | --- | --- | --- | --- | --- |
|  |  | N | M | SD | % “Not at all”*^b^* | % “Very much”^b^ | *% Miss^c^* |
| RELSHP+ | …I appreciate relationships and people much more. | 9,810 | 3.11 | 1.09 | 10.1 | 6.8 | *6.8* |
| PHYS+ | …I pay more attention to my health. | 10,111 | 3.51 | 0.99 | 3.5 | 13.2 | *4.0* |
| COGN+ | …I have more experience and knowledge to evaluate things and people. | 9,961 | 3.07 | 1.01 | 7.7 | 4.4 | *5.6* |
| COGN-EMOT+ | …I have a better sense of what is important to me. | 9,981 | 3.41 | 1.01 | 5.7 | 8.8 | *5.2* |
| ENGAGE+ | …I have more freedom to live my days the way I want. | 10,129 | 2.98 | 1.19 | 15.3 | 7.6 | *4.0* |
| COGN- | …my mental capacity is declining. | 10,181 | 2.52 | 1.06 | 15.7 | 4.9 | *3.5* |
| ENGAGE- | …I have to limit my activities. | 10,211 | 3.39 | 1.14 | 4.2 | 19.3 | *3.2* |
| PHYS- | …I have less energy. | 10,195 | 3.28 | 1.06 | 3.4 | 13.5 | *3.4* |
| RELSHP- | …I feel more dependent on the help of others. | 10,198 | 2.75 | 1.32 | 20.2 | 12.1 | *3.4* |
| COGN-EMOT- | …I find it harder to motivate myself. | 10,155 | 2.63 | 1.12 | 17.4 | 5.3 | *3.7* |

Note. Weighted data.

^a^ Awareness of Age-Related Change Domain Abbreviations: PHYS = Health and Physical Functioning; COGN = Cognitive Functioning; RELSHP = Interpersonal Relations; COGN-EMOT = Social-Cognitive and Social-Emotional Functioning; ENGAGE = Lifestyle and Engagement. ‘+’ positive domains; ‘-’ = negative domains.

^b^ Percentage of extreme categories based on available substantive responses.

^c^ Overall percentage of item nonresponse.

Supplementary Table S3.

*Correlations Between Study Variables.*

| Pearson correlation [95% CI] | | 1 | 2 | 3 | 4 | 5 | 6 | 7 |
| --- | --- | --- | --- | --- | --- | --- | --- | --- |
| 1 | Chronological age (80-106) | 1 |  |  |  |  |  |  |
| 2 | Autonomy (1-4) | **-.22 [-.25 – -.19]** | 1 |  |  |  |  |  |
| 3 | Functional health (IADL 0-2) | **-.44 [-.46 – -.41]** | **.52 [.50 – .54]** | 1 |  |  |  |  |
| 4 | Depressive symptoms (DIA-S4, 0-4) | **.15 [.13 – .17]** | **-.37 [-.40 – -.35]** | **-.42 [-.44 – -.39]** | 1 |  |  |  |
| 5 | Multimorbidity (0-19) | **.11 [.09 – .13]** | **-.20 [-.23 – -.18]** | **-.33 [-.35 – -.31]** | **.33 [.31 – .35]** | 1 |  |  |
| 6 | Education (ISCED, 1-8)^a^ | **-.12 [-.14 – -.10]** | **.10 [.08 – .12]** | **.19 [.17 – .21]** | **-.13 [-.16 – -.11]** | **-.07 [-.10 – -.05]** | 1 |  |
| 7 | AARC-Gains (1-5) | **-.16 [-.18 – -.14]** | **.26 [.24 –.29]** | **.26 [.23 –.28]** | **-.14 [-.17 – -.12]** | .01 [-.02 – .03] | **.10 [.08 – .12]** | 1 |
| 8 | AARC-Losses (1-5) | **.31 [.29 – .33]** | **-.43 [-.45 – -.40]** | **-.64 [-.66 – -.62]** | **.49 [.47 – .51]** | **.38 [.36 – .40]** | **-.12 [-.14 – -.10]** | **-.13 [-.15 – -.10]** |

Note. ISCED = International Standard Classification of Education; IADL = Instrumental Activities of Daily Living; DIA-S4 = Depression in Old Age Scale with four items; AARC = Awareness of Age-Related Change. Weighted data.

^a^ For correlational analysis, we used the eight-category version of the educational achievement variable available in the D80+ study. Correlations statistically significant at the p<.05 level are given in bold font.

Supplementary Table S4.

*Nested Model Comparison of Configural, Metric and Scalar Measurement Invariance (MI) for the AARC-10SF Across Survey Modes (Questionnaire vs Phone Interview).*

| Degree of invariance  (equality constraint) | Absolute model fit | Relative fit indices | LR difference test^a^ |
| --- | --- | --- | --- |
| Configural MI (factor model) | χ^2^ = 1758.2, df = 68, p < .001 | RMSEA(CI90) = 0.069 (0.066-0.072), p < .001 |  |
| Metric MI (loadings) | χ^2^ = 1596.7, df = 76, p < .001 | RMSEA(CI90) = 0.062 (0.059-0.064), p < .001 | Δ-2LL_corr_ = 13.07, Δdf = 8, p = .110 |
| Scalar MI (loadings and intercepts) | χ^2^ = 1548.7, df = 84, p < .001 | RMSEA(CI90) = 0.058 (0.055-0.060), p < .001 | Δ-2LL_corr_ = 22.71, Δdf = 16, p = .122 |

Note. ^a^Likelihood-ratio model comparison for robust maximum likelihood estimation used scaling correction factors.

Supplementary Table S5.

*Domain-level perceived AARC-Gains and AARC-Losses in Community-Dwelling Older Adults and Nursing Home Residents.*

| Domain^a^ | With my increasing age, I realize that … | *Community-dwelling adults (N=9,991)* | | *Nursing home residents (N=587)* | | |
| --- | --- | --- | --- | --- | --- | --- |
|  |  | Mean | 95% CI | Mean | | 95% CI |
| RELSHP+ | …I appreciate relationships and people much more. | 3.13 | [3.10­–3.15] | 3.00 | [2.89–3.12] | |
| PHYS+ | …I pay more attention to my health. | **3.55** | **[3.53­–3.57]** | **3.14** | **[3.02­–3.26]** | |
| COGN+ | …I have more experience and knowledge to evaluate things and people. | **3.13** | **[3.11­–3.16]** | **2.55** | **[2.44–2.66]** | |
| COGN-EMOT+ | …I have a better sense of what is important to me. | **3.46** | **[3.44­–3.49]** | **2.91** | **[2.79­–3.03]** | |
| ENGAGE+ | …I have more freedom to live my days the way I want. | **3.08** | **[3.05­–3.10]** | **2.12** | **[2.00­–2.24]** | |
| COGN- | …my mental capacity is declining. | **2.43** | **[2.40­–2.45]** | **3.33** | **[3.19­–3.47]** | |
| ENGAGE- | …I have to limit my activities. | **3.32** | **[3.29­–3.35]** | **4.03** | **[3.93­–4.14]** | |
| PHYS- | …I have less energy. | **3.21** | **[3.19­–3.24]** | **3.89** | **[3.78­–4.00]** | |
| RELSHP- | …I feel more dependent on the help of others. | **2.61** | **[2.58­–2.65]** | **3.96** | **[3.84­–4.08]** | |
| COGN-EMOT- | …I find it harder to motivate myself. | **2.54** | **[2.52­–2.57]** | **3.33** | **[3.21­–3.46]** | |

Note. Weighted data.

^a^AARC Domain Abbreviations: PHYS = Health and Physical Functioning; COGN = Cognitive Functioning; RELSHP = Interpersonal Relations; COGN-EMOT = Social-Cognitive and Social-Emotional Functioning; ENGAGE = Lifestyle and Engagement. ‘+’ positive domains; ‘-’ = negative domains. Subgroup differences statistically significant at the p<.05 level (non-overlapping 95% confidence intervals) are given in bold font.


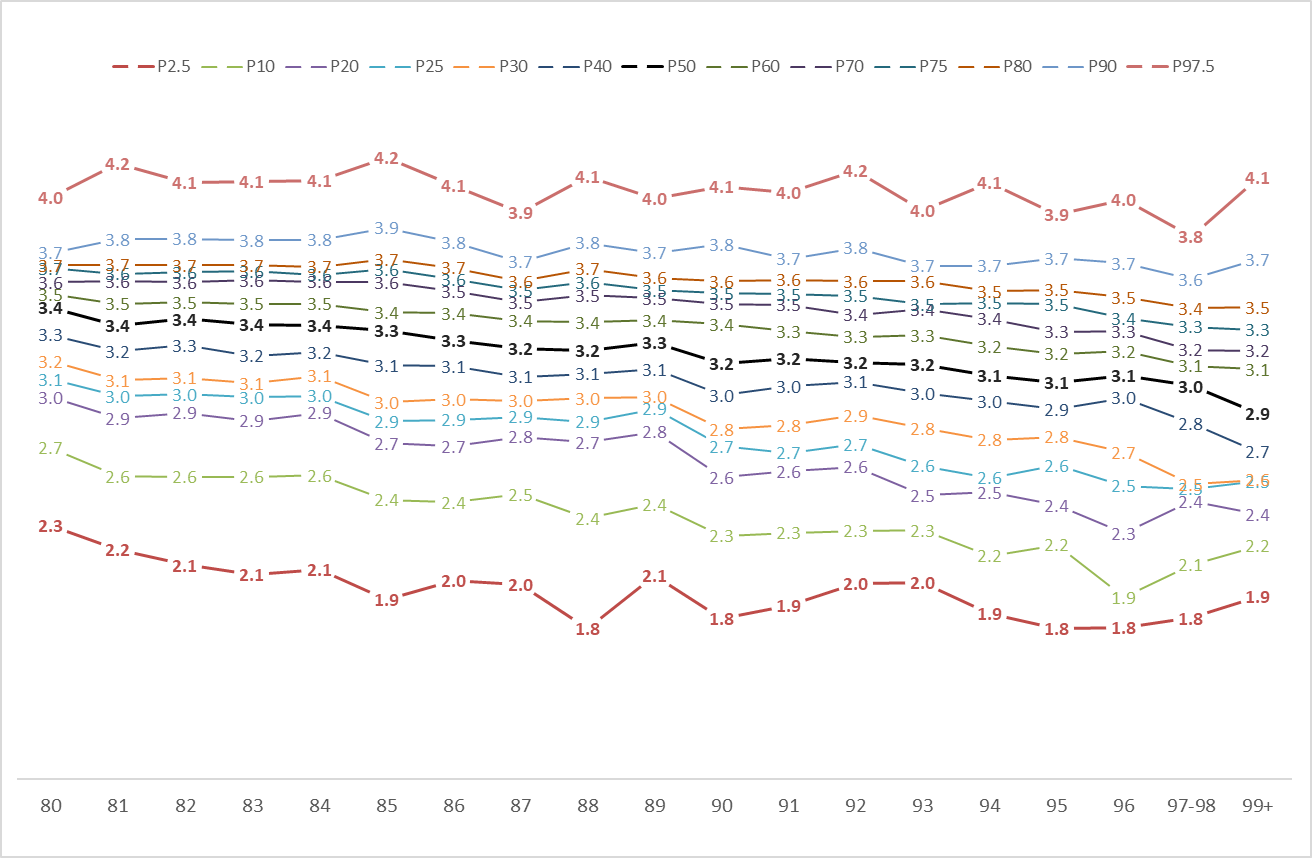


**AARC-Gains**

Chronological age

Percentile:

Supplementary Figure S1.

*Rank Percent of AARC-10SF Gains Scores by Age Cohort.* In the age 80 population, 50% of individuals (i.e. bold median line P50) obtain values of 3.4 or less on the gains scale.


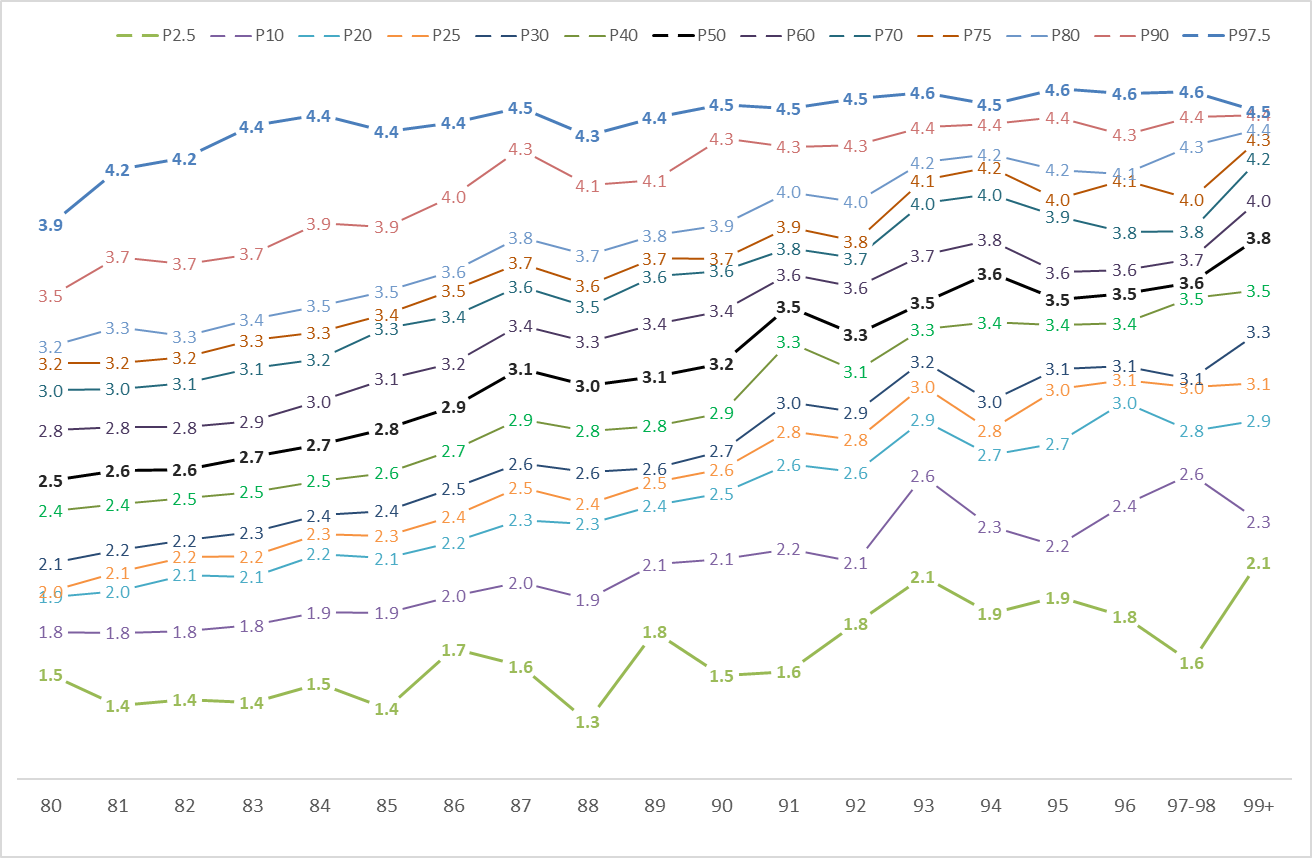


**AARC-Losses**

Chronological age

Percentile:

Supplementary Figure S2.

*Rank Percent of AARC-10SF Losses Scores by Age Cohort.* In the age 80 population, 50% of individuals (i.e. bold median line P50) obtain values of 2.5 or less on the losses scale.

| 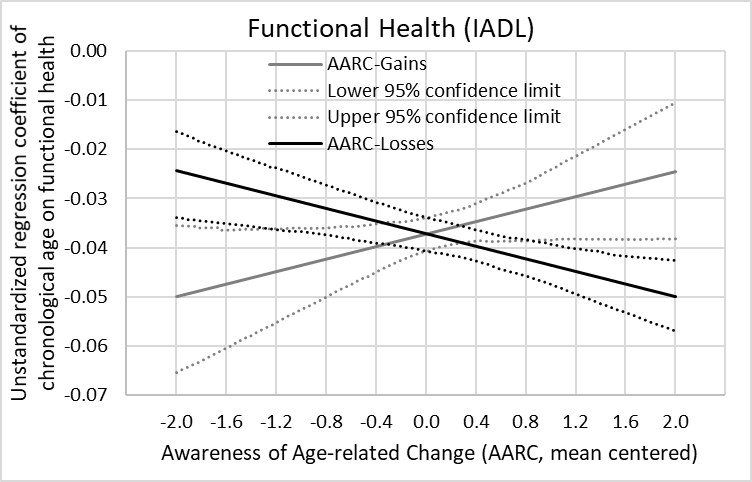 | 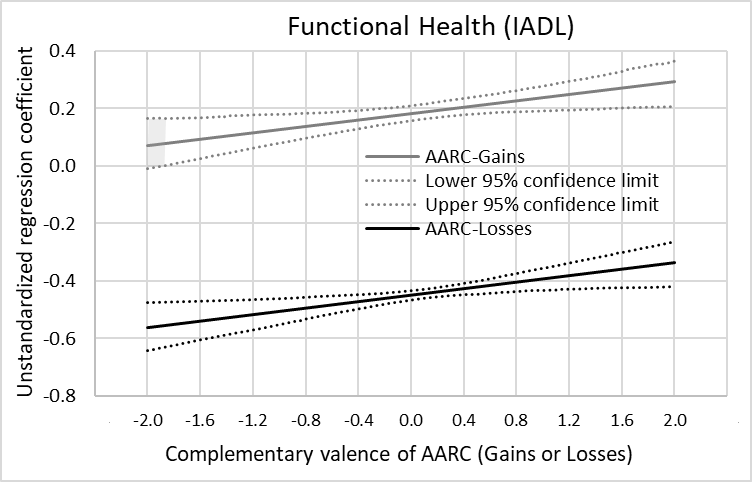 |
| --- | --- |
| 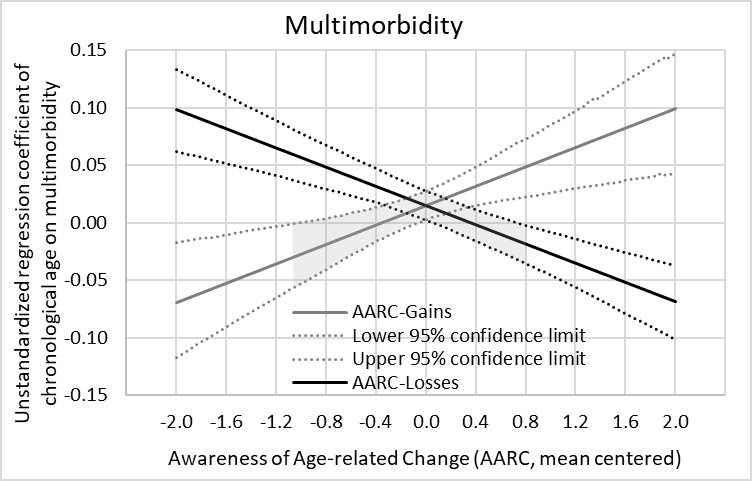 | 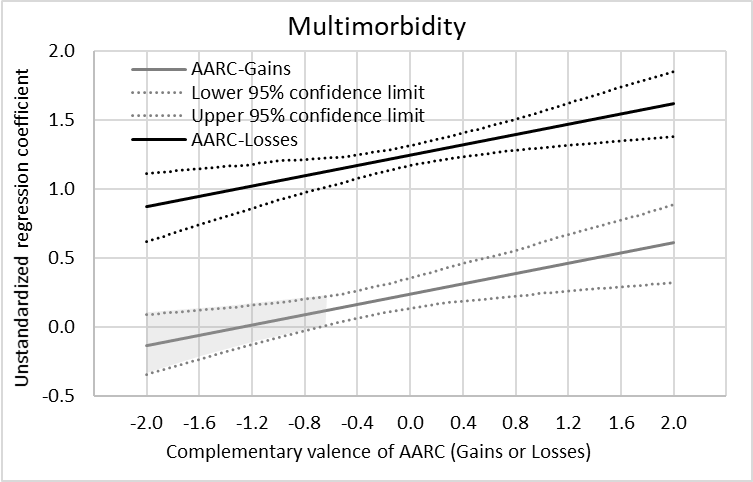 |
| 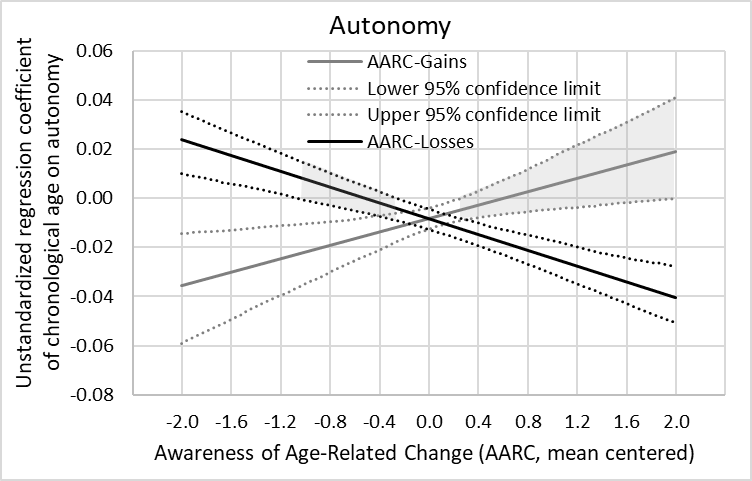 | 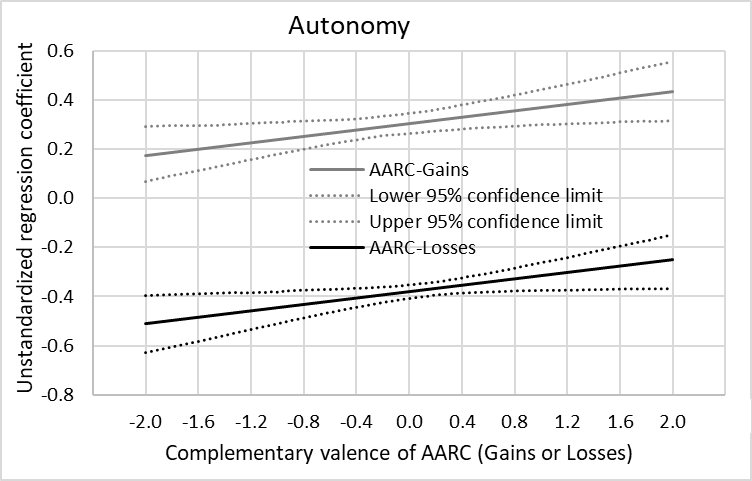 |
| 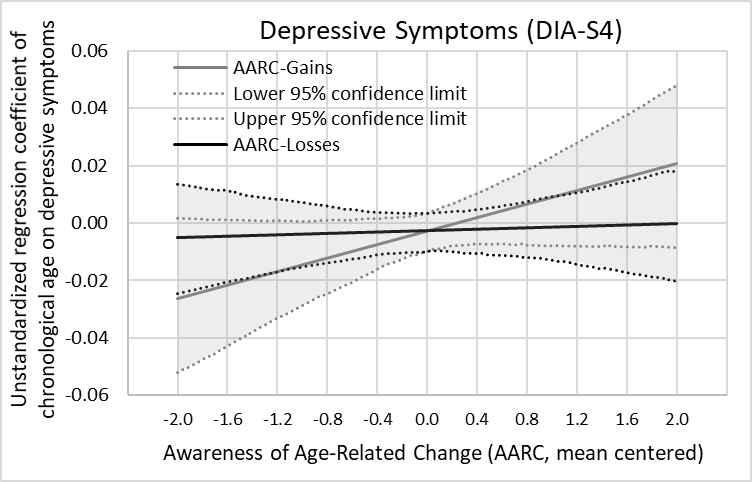 | 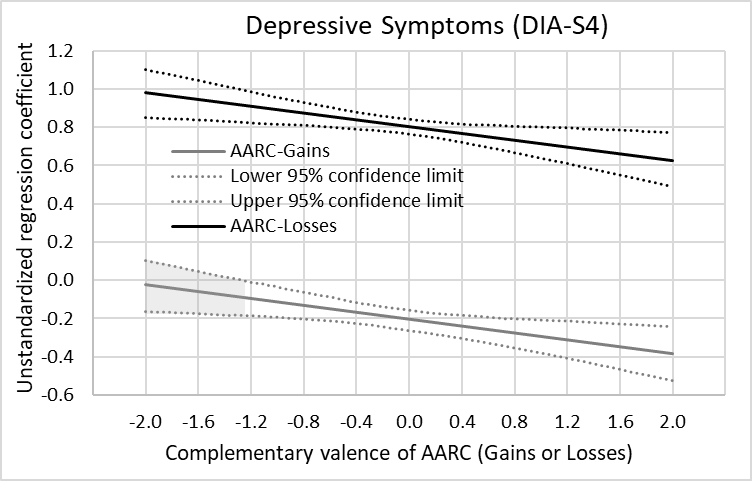 |

Supplementary Figure S3.

*Moderation of the Health-Chronological Age Association by AARC-Gains and AARC-Losses (left panel) and Effects of AARC Gains-to-Losses Balance in Predicting Late-Life Health Correlates (right panel).*
